# Revealing the Roles of *MOAP1* in Diseases: A Review

**DOI:** 10.3390/cells11050889

**Published:** 2022-03-04

**Authors:** Yanfang Su, Weixia Wang, Xianfang Meng

**Affiliations:** Department of Neurobiology, School of Basic Medical Sciences, Tongji Medical College, Huazhong University of Science and Technology, Wuhan 430030, China; m202075309@hust.edu.cn (Y.S.); u201810265@hust.edu.cn (W.W.)

**Keywords:** *MOAP1*, cancer, neurodegenerative diseases, apoptosis, biomarker

## Abstract

Modulator of apoptosis protein1 (*MOAP1*), also known as *MAP1* and *PNMA4*, belongs to the PNMA gene family consisting of at least 15 genes located on different chromosomes. *MOAP1* interacts with the BAX protein, one of the most important apoptosis regulators. Due to its critical role in a few of disease-associated pathways, *MOAP1* is associated with many diseases such as cancers and neurological diseases. In this study, we introduced *MOAP1* and its biological functions and reviewed the associations between *MOAP1* and a few diseases including cancers, neurological diseases, and other diseases such as inflammation and heart diseases. We also explained possible biological mechanisms underlying the associations between *MOAP1* and these diseases, and discussed a few future directions regarding *MOAP1*, especially its potential roles in neurodegenerative disorders. In summary, *MOAP1* plays a critical role in the development and progression of cancers and neurological diseases by regulating a few genes related to cellular apoptosis such as *BAX* and *RASSF1A* and interacting with disease-associated miRNAs, including miR-25 and miR1228.

## 1. Introduction

The modulator of apoptosis protein1 (*MOAP1*), also known as *MAP1* and *PNMA4*, belongs to the *PNMA* gene family, which consists of at least 15 genes on different chromosomes [1]. Specifically, *PNMA1* and *PNMA4* are located in human chromosome 14; *PNMA8A*, *PNMA8B*, *PNMA8C*, and *CCDC8* (a paralog of *PNMA6F*) are localized to chromosome 19; *PNMA2* belongs to chromosome 8; and *PNMA3*, *PNMA5*, *PNMA6A*, *PNMA6B*, *PNMA6E*, *PNMA6F*, *PNMA7A*, and *PNMA7B* are located in X chromosome [1]. It is known that the PNMA family is highly expressed in the brain, testis, and heart with MOAP1, a resident protein in the mitochondria [1]. There are multiple acronyms existing for *MOAP1* (e.g., *MAP1*, *PNMA4*, etc.), and we unified them to *MOAP1* from now on to avoid any confusion.

Among the *PNMA* genes, *MOAP1* is probably the most important because it not only plays a central role in mitochondria and death receptor-mediated apoptosis [2], but also is associated with many diseases like cancer [3]. The specific roles of *MOAP1* in cellular apoptosis and various diseases are determined by its protein structure. Specifically, in the BH3L domain of *MOAP1*, a unique protein sequence or structure (UPD) between the NCD and CCD domains was identified. The protein sequence of *MOAP1* also contains a domain rich in lysine and arginine residues, named KRs [1]. 

First identified by the yeast-two-hybridization assay, MOAP1 was a BAX-associated protein that binds to BAX only during apoptosis induction [2]. It plays a vital role in mitochondrial-dependent apoptosis by influencing and maintaining BAX activation. The down-regulation of MOAP1 leads to the instability and polymerization of BAX on the mitochondrial membrane so that cytochrome C is not released into the cytoplasm. The knockdown of this protein inhibited the apoptotic stimulation induced by TNFα and TRAIL. Therefore, abolishing this protein will cause cells to be insensitive to death signals mediated by the external and internal pathways of apoptosis [4]. 

In addition, the complement system is a tightly regulated network of proteins involved in defense against pathogens, inflammatory processes, and the coordination of innate and adaptive immune responses. Hertz et al. mentioned *MOAP1* as a pattern-recognition molecule (PRM) related to inhibitors of the complement system’s lectin pathway. The C4B binding protein (C4BP) regulates both classical and lectin pathways and MOAP1/C4BP chimeras have unique complement inhibition properties, providing a new therapeutic approach for upstream and central complement activation [4]. *MOAP1* also plays a role in liver Fas signaling by promoting MTCH2-mediated tBid recruitment to mitochondria [5].

Besides BAX, *MOAP1* also attenuates cell apoptosis by collaborating with other molecules. For example, it is known that *RASSF1A* and *MOAP1* intimately interact to form the *RASSF1A*/*MOAP1* molecular pathway in cancer. One potential function of this pathway is to link death receptors with BAX conformational change to promote tumor suppression [6]. *MOAP1* also interacts with *RASSF6* to attenuate *RASSF6*-induced cell apoptosis; however, the attenuation was not observed in a p53-negative background [7,8,9].

Moreover, *MOAP1* is regulated by ubiquitin protein degradation [10]. Ubiquitin proteasome system is an indispensable process during the transformation from normal cells to cancer cells. There are a few studies showing that a new regulation factor, *Trim39,* can stabilize *MOAP1* [11,12]. Moreover, *UBR5* enhances *MOAP1* ubiquitination. Matsuura et al. found that *UBR5* ubiquitin ligase down-regulates *MOAP1* in ovarian cancer, which contributes to its role in ovarian cancer resistance to cisplatin [13]. Since the in-vitro *MOAP1* expression level is low in tumor cells, *MOAP1* ubiquitin can be reduced by apoptosis stimulating processes [13]. 

Finally, there are a few studies suggesting a unique role of *MOAP1* in promoting autophagy signal transduction. Autophagy goes through three different stages by phagocytotic molecules and organelles, including initiation of recruitment, nucleation, and expansion of phagocytic membrane, followed by lysosomal degradation. P62 is an autophagy receptor. The BAX-binding protein *MOAP1* regulates the p62-KEAP1-NRF2 signaling pathway by disrupting the p62 corpuscles. *MOAP1* is recruited to p62 corpuscles by the induction of formation stimulated by cellular stress and reducing their levels independently of the autophagy pathway [14]. Interestingly, *MOAP1* was found to be an LC3-binding protein, which plays a unique role in promoting autophagy by interacting with LC3 to promote efficient shutdown of autophagy during starvation [15].

**Figure 1:**
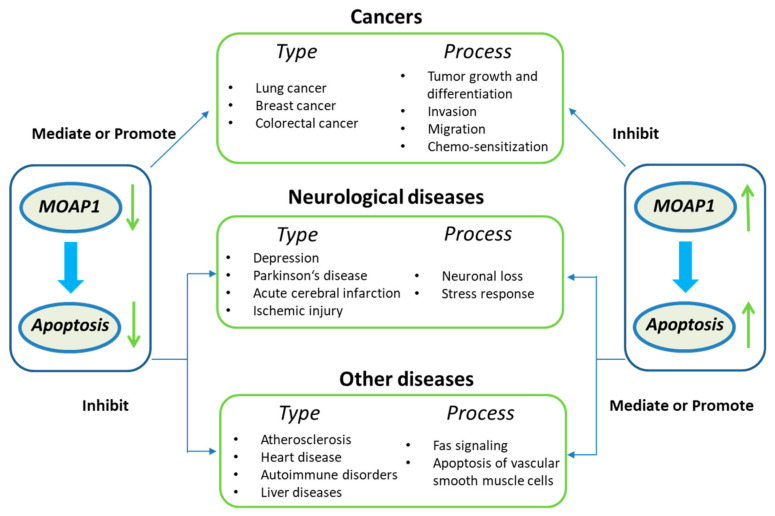
The common functions of *MOAP1* and its related diseases.

In summary, *MOAP1* can activate the apoptosis signaling pathway, promoting its role in many diseases, such as cancers and neurological diseases (Figure 1). For example, *MOAP1* is involved in the progression, invasion, metastasis, and chemo-sensitization of various cancers, including lung cancer, colorectal cancer, and breast cancer [16,17,18]. In the nervous system, *MOAP1* can induce neuronal loss, actuate cerebral infarction, depressive behavior, and stress response [19,20]. Finally, Fas, also known as Cd95 and *APO1*, is involved in diseases like atherosclerosis, heart disease, autoimmune disorders, liver disease, and myocardial infarction [21,22,23,24,25]. Since there is evidence that *MOAP1* and Fas signaling are highly associated [5], *MOAP1* may also play a role in these diseases. In the following, we will review the molecules mediating the association between *MOAP1* and the diseases mentioned above and their acting mechanisms. 

## 2. Roles of *MOAP1* in Cancers

With the increasing of human lifespan, cancer has become one of the top three diseases threatening human lives [26]. The hallmarks of cancer, including evading apoptosis, self-sufficiency in growth signals, insensitivity to anti-growth signals, tissue invasion and metastasis, limitless replicative potential, and sustained angiogenesis; the five processes are mutually interacted [27]. Previous studies have suggested that *MOAP1* is highly associated with the apoptosis pathway [28], one of the most critical pathways and hallmarks in cancer. Thus, the roles of *MOAP1* in various cancers and related biological mechanisms have been widely studied [16,29], among which lung cancer, colorectal cancer (CRC), and breast cancer are probably the most well studied. 

### 2.1. MOAP1 in Lung Cancer

Lung cancer is the most prevalent cancer in China and the third most common cancer in the United States (https://www.cdc.gov/cancer/lung/ accessed on 9 December 2021), accounting for approximately 13% of new cancer incidences worldwide and causing 1.4 million deaths each year [30]. Lung cancer consists of small cell lung cancer (SCLC) and non-small cell lung cancer (NSCLC), which can be further divided into squamous-cell carcinoma, adenocarcinoma, large-cell carcinoma, and so on. 

**Figure 2:**
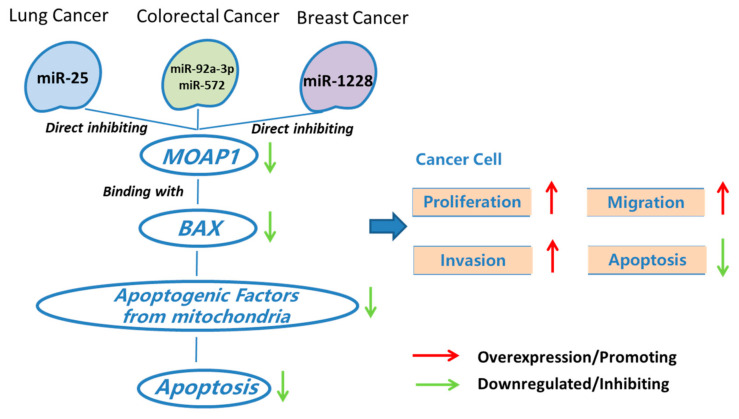
*MOAP1* acts as a modulator on cancer-associated miRNAs and cancers.

The proteins in the BCL2 family can regulate the intrinsic apoptotic pathway [31]. BCL2 family proteins consist of pro-apoptotic proteins (like BAX, BAK, and BAD) and anti-apoptotic proteins (like BCL2, BCLW, and MCL1). Liu et al. observed abnormal expression of BCL2 protein in NSCLC and showed that BCL2 and MCL1 amplification is related to drug resistance [32]. Chan et al. found that MOAP1 can directly or indirectly interact with BCL2 family proteins, which promotes its role in lung cancer [15] (Figure 2). 

On the other way, the role of miRNAs in regulating the apoptosis pathway in lung cancer has been widely studied. For example, some miRNAs are critical in lung cancer treatment by targeting TP53-dependent cell apoptosis [33]. Previous studies also suggested that miRNAs regulating lung cancer cell apoptosis interact with many important cancer pathways, including PI3K/Akt, PTEN, GSK-3b, NF-kB, BCL2, BAX, P53, and mTOR [34]. However, the biological mechanisms underlying lung cancer-associated miRNAs are unknown. *MOAP1* is one of the genes to mediate lung cancer-related miRNAs and lung cancer. That is, a few miRNAs can regulate *MOAP1* and BAX, which may contribute to the apoptosis of lung cancer cells. For example, miR-25 is a critical biomarker for non-small-cell lung cancer. Wu et al. found that this miRNA down-regulates the expression of *MOAP1*, which promotes cell proliferation and inhibits caspase-dependent apoptosis in NSCLC cells (Figure 2). Thus, miR-25 antagomir can promote *MOAP1* expression and thus inhibit lung cancer growth in a mouse xenograft model [16]. We refer to synthetic antagonists as silencing agents of miRNAs by the term antagomir.

### 2.2. MOAP1 in Colorectal Cancer

Colorectal cancer (CRC), also known as bowel cancer, is among the top three most common cancers in the world. This cancer is caused by uncontrolled cell growth in the colon or rectum; the tumors in the two body parts are genetically the same and thus are called uniformly as colorectal cancer (https://www.nature.com/subjects/colorectal-cancer accessed on 9 December 2021). One major issue in CRC and probably all cancers is recurrence and metastasis, which eventually leads to the death of most cancer patients. That is, the cancer cell re-grows in the same location after surgical resection or move to another body location through blood flow and proliferation [35]. 

In recent years, much effort has been placed on revealing the biological mechanisms behind the progression, recurrence, and metastasis of CRC. Among many pathways related to CRC, p53-dependent apoptosis is probably the most studied. BAX and BCL2 are two important regulators of p53-dependent apoptosis and thus are critical in many processes of CRC. For example, Katkoori et al. suggested that the expression level of BAX in a CRC patient can be used to predict his/her prognosis [36]. Low BAX expression was considered a negative prognostic factor for CRC patients with liver metastasis in an early study [37]. The BAX/BCL2 ratio was also considered one of the prognostic markers for tumor location in CRC [38]. Besides the prognostic value, BAX and BCL2 are also important for CRC at molecular levels. Pryczynicz et al. found that BAX protein could reduce CRC invasion [38]. Jansson and Sun found that the expression of BAX was significantly higher in metastatic CRC than in primary CRC [39]. In addition, BAX is also involved in other inhibition mechanisms associated with CRC, such as the PGC1α pro-apoptotic effect [40], regulating autophagy-related genes [41], and mediating CRC with microsatellite instability [42]. Similarly, BCL2 is a suppressor of p53-dependent apoptosis in CRC [43] and is found to regulate the invasion and metastasis of CRC cells [44].

It is known that BAX could be regulated by *MOAP1* in CRC [3,6], which promotes the roles of *MOAP1* in various processes of CRC (Figure 2). Specifically, the function of BAX and *MOAP1* might be regulated by a few miRNAs. For instance, miR-92a-3p and miR-572 are two known colorectal cancer-associated miRNAs. High miR-92a-3p expression contributes to chemotherapy resistance and metastasis of CRC [17]. A recent study suggested that these functions of miR-92a-3p were achieved by inhibiting *FBXW7* and *MOAP1* directly to activate the Wnt/β-catenin pathway and inhibit cell apoptosis, respectively [17] (Figure 2). In addition, the up-regulation of miR-572 can inhibit *MOAP1* expression, which promotes the migration, invasion, and growth of CRC cells [29] (Figure 2). As a result, miR-572 antagomir might be a potential therapeutic means for treating colorectal cancer. 

### 2.3. MOAP1 in Breast Cancer

Breast cancer is the second most common woman cancer in the United States (https://www.cdc.gov/cancer/breast/statistics/ accessed on 9 December 2021). There are five main molecular subtypes of breast cancer: Luminal A, Luminal B, Triple-negative/basal-like, HER2-enriched, and Normal-like. HER2 is probably the most important gene for breast cancer, with HER2-positive and HER2-negative patients accounting for about 25–30% and 70–75% of all breast cancer incidences, respectively [45,46,47]. Even after trastuzumab adjuvant treatment, a large portion of breast cancer develops recurrence and metastasis, especially for the HER2-positive subtype. 

The role of BAX apoptosis signaling in breast cancer has been widely studied. BAX and BCL2 are known prognostic biomarkers for breast cancer. *BCL2*, also known as BCL2 Apoptosis Regulator and B-cell lymphoma-2, is a gene capable of suppressing apoptosis. Specifically, the integral outer mitochondrial membrane protein encoded by *BCL2* can block the apoptotic death of many cells like lymphocytes, making it a prognostic biomarker for cancers. Sjostrom et al. identified the predictive value of BAX and BCL2 in chemotherapy responses in advanced breast cancer [48]. Azimian et al. found that the ratio between the expressions of BAX and BCL2 (BAX/BCL2) could be used to predict the effect of breast cancer radiotherapy in an individual patient [49]. In addition, Binder et al. found that the differential BAX/BCL2 expression pattern is important for cancer cell proliferation and thus is a predictive marker for cancer malignant progression [50]. Linjawi et al. identified that BCL2 expression is significantly correlated with hormonal receptors in breast cancer patients. Sehrawat suggested that BAX activation can mediate the selective death of human breast cancer cells and might provide some direction for breast cancer treatment. Finally, a recent study suggested that the tricistronic expression of *MOAP1*, BAX, and *RASSF1A* enhances chemo-sensitization in breast cancer cell lines [51].

As a result, *MOAP1,* its upstream genes, and miRNAs play critical roles in the progression, invasion, and migration of breast cancer. For example, miR-1228 is a miRNA responsible for breast cancer invasion and migration. Lin et al. found that miR-1228 could be attenuated by increasing the expression of *MOAP1* (Figure 2), suggesting the potential of *MOAP1* in breast cancer studies and therapies. 

Together, the downregulation of *MOAP1* could reduce the effect of BAX, which may suppress p53-dependent cancer cell apoptosis. This process places an important role in bridging various cancers and their associated miRNAs. Overexpression of these miRNAs usually inhibits the expression of *MOAP1*, thus promoting cancer cell proliferation, migration, and invasion. 

### 2.4. MOAP1 in Cancer Drug Resistance and Chemo-Sensitization

**Figure 3:**
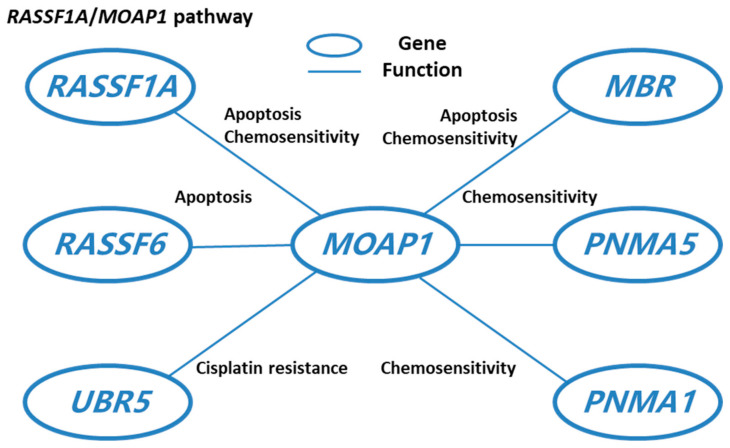
*MOAP1* interacts with other genes to perform cancer-related functions and the literature supporting the interactions and functions.

*MOAP1* was involved in cancer drug resistance and chemo-sensitization (Figure 3). For example, Matsuura et al. found that *UBR5* down-regulates proapoptotic *MOAP1* in ovarian cancer, which results in cisplatin resistance [13] (Figure 3). In cancer cells, tricistronic expression of *MOAP1*, BAX, and *RASSF1A* (MBR) expression will induce cell death and thus increase chemo-sensitization. Recently, Lee et al. found that this process requires the involvement of the BH3L domain of *MOAP1* [51]. As a member of the *PNMA* family, *MOAP1* also interacts with other members in this family to promote chemo-sensitization. In MCF-7 cells, overexpression of *MOAP1* and *PNMA1* will promote chemo-sensitization; however, the chemo-sensitization will be greatly inhibited when co-expressing with *PNMA2*, indicating that *PNMA2* might be an antagonist of *MOAP1* and *PNMA1* induced chemo-sensitization [52]. In addition, the co-expression of *PNMA5* and *MOAP1* greatly enhances the chemo-sensitivity of MCF-7 cells to Etoposide treatment [52].

## 3. Roles of *MOAP1* in Neurological Diseases

Previous studies suggested that *PNMA2*, *PNMA3*, and *MOAP1* mRNAs are abundantly expressed in mouse brains. In the neocortex, *PNMA2*, *PNMA3*, and *MOAP1* mRNAs are expressed throughout all layers, although the expression levels of the genes differ slightly among the layers [19]. It is known that neurological diseases, especially neuron-degenerative diseases such as Alzheimer’s disease and Parkinson’s disease, are highly associated with mitochondrial-dependent neuron cell apoptosis [53,54]. In addition, *MOAP1* is abundantly expressed in other brain tissues [20,55]. *MOAP1* is speculated to be associated with neurological diseases, and there are a few studies in this area. 

### 3.1. MOAP1 and Cerebral Ischemia

Stroke remains one of the top diseases for human disability and death worldwide. This is largely due to the narrow time window for recognition and the administration of outcome-modifying treatment [56]. Acute stroke can result in permanent damage to the human brain and even cognitive death, making it critical to unveil the mechanism behind its formation and treatments. 

A few early studies suggested that upregulation of BAX proteins in neuronal cells is a risk factor for cerebral ischemia [57,58]. BAX ablation might be a feasible direction to control cerebral ischemia [59], which makes *MOAP1* critical in cerebral ischemia since it can regulate BAX and BCL2 in neurons. Studies have shown that the *MOAP1−/−* primary cortical neurons and *MOAP1*þ/þ primary neurons are resistant against oxygen and glucose deprivation (OGD) treatment; *MOAP1−/−* primary cortical neurons are stronger. In the mouse transient middle cerebral artery occlusion (tMCAO) model, cerebral ischemia causes the *MOAP1*/BAX association, activating the *MOAP1*-dependent apoptosis cascade. In the study, the authors found that 24 h after tMCAO, *MOAP1−/−* mice showed less neuron loss and smaller infarct volume than *MOAP1+/+* mice. Similarly, *MOAP1−/−* mice also have better integrity in neurological functions, which was proved in their rotarod test [20]. Together, these studies suggested that *MOAP1* plays an important role in regulating apoptosis in cerebral ischemic injury.

### 3.2. MOAP1 and Depression 

Stress response is closely related to one of the most important mental diseases, namely depression. Depression, also called major depressive disorder, is a serious mental illness that negatively affects people’s the feelings and actions. 

Depression and BAX apoptosis signaling are highly correlated. For example, the signal cascade composed of BAD, BAX, and Caspase 3 is sufficient for synaptic depression [60]. In addition, the upregulation of BAX and downregulation of BCL2 were observed in the olfactory bulb of a rat depression model [61]. Due to its regulatory role in BAX apoptosis, *MOAP1* plays an important role in regulating stress response by the Dorsal raphe nucleus (DRN). Forced swimming test is one of the famous techniques in wet lab to develop depression model in mice [62]. Compared with age-matched wild-type mice, young *MOAP1*−/− mice in the forced swimming test showed depressive behavior with increased stationary time, which was eliminated by acute fluoxetine treatment. In young wild-type (WT) control mice, repeated forced swimming stress resulted in upregulation of tryptophan hydroxylase 2 (*TPH2*) and down-regulation of brain-derived neurotrophic factor (*BDNF*) in the dorsal renal nucleus (DRN). In contrast, *TPH2* was not upregulated in aging WT mice. Interestingly, this stress response was not present in both young and old *MOAP1*−/− mice [55]. 

### 3.3. MOAP1 and Parkinson’s Disease 

Parkinson’s disease (PD) becomes the second most common neurodegenerative disease in the world after Alzheimer’s disease, affecting nearly five million people worldwide, and this number is expected to double by 2030 [63]. In Parkinson’s disease, the main pathological features are connected with progressive neurodegeneration of dopaminergic neurons and typical motor characteristics in the substantia nigra striatum (SNC), which locates in the dense region of the black substantia [64]. It results from genetic, environmental, and aging factors and currently has no good treatment. Apoptosis and autophagy play a vital function in the pathogenesis of PD. Therefore, adjusting their balance is a potential therapeutic strategy [63]. Previous studies have suggested that the BCL2 protein family is a therapeutic biomarker in Parkinson’s disease [53,65,66,67]. Specifically, proteins in the BCL2 family, such as BAX, can mediate the apoptosis of dopaminergic neurons in the Parkinson’s disease. For example, Vila et al. found that the ablation of BAX protein can prevent neurodegeneration in Parkinson’s disease [68]. By targeting BAX, some miRNAs like MicroRNA-216a also inhibit neuronal cell apoptosis in Parkinson’s disease [69]. Thus, proteins in the BCL2 family might be a promising target in treating Parkinson’s disease.

It is well known that *MOAP1* directly binds to BAX, which suggests that *MOAP1* may play a role in Parkinson’s disease. Capurro et al. used external laser capture micro-anatomical data to verify the differentially expressed genes identified by PSEA (population-specific expression analysis) in the dense part of substantia nigra neurons. In this study, the expression of the *MOAP1* gene was found to be reduced in cortical neurons of Parkinson’s disease (PD) [70]. In Table 1, we summarized the genes involved in the connection between *MOAP1* and neuron-related traits and diseases. Besides BAX, *MOAP1* also interacts with other molecules like *PNMA2*. For example, in immunoprecipitation studies, *PNMA2* was closely related to *PNMA1* and *MOAP1* and functionally antagonized the pro-apoptotic of *MOAP1* and *PNMA1* [71].

Besides cancers and neuronal diseases, *MOAP1* was also related to a few other diseases. For example, *MOAP1* was targeted by miR-25 to block the apoptosis of vascular smooth muscle cells, which plays a critical role in inflammation and heart disease [21]. *MOAP1* is also commonly identified differential genes of coronary artery disease, ischemic cardiomyopathy, and myocardial infarction [73,74]. In addition, it is known that Fas, also known as Cd95 and Apo1, is implicated in a few diseases like liver diseases, autoimmune disorders, and fulminant hepatitis [22,23,24,25]. Tan et al. found that *MOAP1* is involved in the Fas signaling for apoptosis in the liver [5] and thus is associated with liver diseases. In summary, *MOAP1* might be implicated in many diseases and its acting mechanisms are yet to be discovered. 

## 4. Conclusions and Future Perspectives on *MOAP1*

In this study, we reviewed the roles and mechanisms of *MOAP1* in a few biological processes including cell apoptosis, innate and adaptive immunity, ubiquitin protein degradation, and autophagy. We also reviewed the molecules mediating the association between *MOAP1* and a few diseases like cancers and neurological diseases. 

As an important gene in the PNMA gene family, *MOAP1* can bind with the BAX protein to activate mitochondria and death receptor-mediated cell apoptosis, which will inhibit the progression and metastasis of a few cancers like lung cancer, breast cancer, and colorectal cancer and may mediate neurological diseases. In addition, a few miRNAs can directly interact with *MOAP1* in a few cancers. For example, miR-25 can directly inhibit *MOAP1* in lung cancer; miR-92a-3p and miR-572 can inhibit *MOAP1* in colorectal cancer, and miR-1228 can inhibit *MOAP1* in breast cancer. These may explain the functions of these miRNAs in cancer etiology. Moreover, *MOAP1* also interacts with other genes to perform specific functions. For example, *MOAP1* and *RASSF1A* collaborate to form the *RASSF1A/MOAP1* pathway, which is important for cell apoptosis and chemosensitivity. *MOAP1* also interacts with *RASSF6* and *MBR* to promote cell apoptosis and is co-expressed with *PNMA5* and *PNMA1* to enhance chemosensitivity.

Interestingly, the role of *MOAP1* in cancers is relatively well studied in the literature; however, its roles in other diseases are yet to be revealed. Other diseases driven by mitochondrial-dependent apoptosis might also have a close relationship with *MOAP1* and need to be further studied. Moreover, it is worthy of mentioning that there are many other biomarkers associated with *MOAP1*, such as long non-coding RNA (lncRNA), DNA methylation, and histone modification, regulating the expression of a gene. For example, the regulating roles of lncRNA in cancer have been widely studied recently [75], and a few lncRNAs such as SNHG1 also play important roles in neuronal cells [76,77]. The co-expression between lncRNA *SNHG5* and *MOAP1* in thyroid cancer has been reported; however, the underlying mechanism is unclear [78]. In the future, studies on the relationship between *MOAP1* and other regulation molecules should be explored.

## Figures and Tables

**Table 1:** The molecules involved in the action of *MOAP1* in neuron-related traits or diseases and their functions.

Gene Symbol	Neuron-Related Traits or Diseases	Function
PNMA2	Abundant in brain	Antagonize the signal transduction of apoptotic cells both *MOAP1* and PNMA1 [71]
PNMA3	Abundant in brain	Gene Ontology (GO) annotations related to this gene include nucleic acid binding (https://www.genecards.org/ accessed on 9 December 2021)
BAX	Ischemic injury	Binding with a novel BAX-related protein (*MOAP1*) mediates apoptosis [2]
TPH2	Depression	Catalytic biosynthesis of serotonin [72]
BDNF	Depression	Bind with its homologous receptor promotes neuronal survival in the adult brain (https://www.genecards.org accessed on 9 December 2021)
